# Falsely positive anti-glomerular basement membrane antibodies in a patient with hantavirus induced acute kidney injury - a case report

**DOI:** 10.1186/s12882-018-1082-3

**Published:** 2018-10-22

**Authors:** H. W. Zijlstra, A. H. L. Mulder, F. Geeraedts, F. Visser

**Affiliations:** 1 Ziekenhuisgroep Twente, Department of Nephrology, Almelo, The Netherlands; 2 Medlon, Department of Clinical Chemistry, Almelo, The Netherlands; 3 Laboratory for Medical Microbiology and Public Health, Hengelo, The Netherlands

**Keywords:** Hantavirus, Anti-GBM glomerulonephritis, Goodpasture’s syndrome, Anti-GBM antibodies, False positive

## Abstract

**Background:**

Hantavirus infection is an uncommon cause of acute renal failure with massive proteinuria. Serology tests to support a presumptive diagnosis usually take a few days. During the initial work-up, autoimmune causes including anti-glomerular basement membrane (GBM) glomerulonephritis need to be excluded, because these require urgent therapy. In this case the delay in serological testing caused a dilemma in treatment initiation.

**Case presentation:**

An 18-year-old patient was admitted to the hospital with acute renal failure, erythrocyturia and massive proteinuria. Routine blood analysis showed leucocytosis (40,5 × 109/l) and a serum creatinine of 233 μmol/l. Infectious causes, e.g. leptospirosis or hantavirus infection, or an autoimmune disease, e.g., AAV or anti-GBM glomerulonephritis was the most feasible underlying diagnosis.

Before hantavirus serology results were known, anti-GBM antibodies were positive. Treatment for anti-GBM glomerulonephritis was withheld, because of the absence of other signs and symptoms of the disease and slight improvement of renal function.

The diagnosis of acute hantavirus infection was later on confirmed, by seroconversion of a follow-up serum sample. Without further intervention renal function recovered and anti-GBM antibodies disappeared.

**Conclusion:**

Hantavirus infection may induce anti-GBM antibodies, falsely suggestive of anti-GBM glomerulonephritis.

Anti-GBM antibodies are supposed to be 100% specific. No earlier reports of false positive anti-GBM titers were reported. Nevertheless, the anti-GBM antibodies in this case were seen as an innocent bystander effect.

Considering the need of urgent initiation of plasmapheresis and administration of immunosuppressants it may lead to diagnostic dilemmas with crucial therapeutic consequences. Knowledge of this anomaly when diagnosing acute renal failure, is very important.

## Background

Hantavirus infection is a relatively rare cause of a possibly life-threatening disease with acute renal failure (haemorrhagic fever with renal syndrome (HFRS) and pulmonary involvement (hantavirus pulmonary syndrome (HPS)). It is a zoonotic infection caused by single-stranded RNA viruses of the hantavirus genus in the family of Bunyaviridae. There are 23 known species within the genus [1]. The most commonly found species in Western-Europe is Puumala virus [2], which is carried by the bank vole (*Myodes glareolus*). Clinical cases of Puumala virus infection usually present as a mild form of HFRS, called nephropathia epidemica (NE). NE normally presents with an influenza-like illness, followed by acute renal failure, with massive proteinuria. Infection occurs by inhalation of aerosols of dried up excretions of the bank vole, containing the virus.

A presumptive diagnosis can be made by testing the patient’s serum for anti-hantavirus antibodies, which usually takes a few days. In case of acute renal failure autoimmune glomerulonephritis has to be excluded immediately, because of the urgency to initiate treatment. These autoimmune diseases include anti-GBM glomerulonephritis and anti-neutrophil cytoplasmic antibody (ANCA)-associated vasculitis (AAV). Treatment needs to be initiated instantly, since the proportion of glomeruli affected at initiation of treatment is a predictor of renal survival [3].

In case of hantavirus infection there is no specific treatment. Generally the treatment is supportive and renal function recovers completely within a few days in most cases. Haemodialysis, oxygen therapy and shock therapy are sometimes needed.

We encountered a case in which the delay in serological results and an unusual laboratory finding lead to a diagnostic dilemma in the first days of admission.

### Case presentation

We present a case of an 18-year-old man with acute renal failure that was admitted to the hospital. The patient, who worked as a chef and reported himself as a smoker,had no known past medical history. He presented with abdominal pain, vomiting and diarrhoea. At presentation he had a tachycardia of 98 beats/min, with a blood pressure of 139/72 mmHg, a body temperature of 36.9 °C and normal urine output. Routine blood analysis showed leucocytosis (40,5 × 10^9^/l (87% neutrophils)) and a serum creatinine of 233 μmol/l, resulting in an estimated glomerular filtration rate of 32 ml/min/1,73 m2, C-reactive protein was 44 mg/l, ALT 32 IU/l, AST 78 IU/l, GGT 25 IU/l, Alkaline Phosphatase 73 IU/l, bilirubin < 17 μmol/l and albumin 24 g/l. Post renal obstruction was excluded using ultrasound sonography. Urine analysis showed erythrocyturia, without casts, and massive proteinuria (protein/creatinine ratio 842.7 mg/mmol creatinine with urine creatinine of 15.7 mmol/l). It was considered as an acute glomerulonephritis, most likely caused by an infectious cause, e.g., leptospirosis or hantavirus infection, or an autoimmune disease, e.g., AAV or anti-GBM glomerulonephritis, considering age, history and physical examination. No specific history indicating hantavirus infection was recorded, e.g., cleaning up dusty sheds, contact with rodents.

ANCA was negative. Anti-GBM was 9.7 kIU/l, which is within equivocal range (7–10 kIU/l).

The detectable anti-GBM antibodies, with hantavirus serology still in progress, lead to a diagnostic and therapeutic dilemma, even though it was still in equivocal range. Treatment of anti-GBM glomerulonephritis consists of plasmapheresis in combination with immunosuppressants, which may be disadvantageous in case of a viral infection and has possible adverse effects. Late initiation of therapy in case of an anti-GBM glomerulonephritis, however, would increase the risk of developing end-stage renal failure [3].

Because of the equivocal anti-GBM antibodies, a slight improvement of renal function on day 2 and no other signs or symptoms of anti-GBM glomerulonephritis, like elevated C-reactive protein or pulmonary involvement, there was a considerable doubt about anti-GBM glomerulonephritis being the correct diagnosis. Therefore no renal biopsy was done and plasmapheresis and immunosuppressants were not administered.

Four days after admission a second anti-GBM measurement showed an increase in anti-GBM antibodies and was undeniably positive (16.0 kIU/l), with further renal and clinical improvement.

Only supportive care was initiated. Dialysis was not needed. The patient’s kidney function improved without further intervention and after 5 days of admission he was discharged from the hospital.

On day seven serum was found positive for anti-Puumala hantavirus IgG as well as IgM antibodies, suggesting acute viral infection.

The spontaneous improvement of renal function is consisting with the natural course of NE and hantavirus infection became the most likely diagnosis. No renal biopsy was done to exclude other causes.

Twelve weeks after admission, renal functions were completely restored and anti-GBM antibodies were no longer detectable. A second serum sample showed a persisting highly.

positive result for anti-Puumala IgG (Ratio 5.2), and a disappearance 118 of IgM antibodies.

119 (Ratio 0.03; < 1.0 is regarded negative) consistent with a completed seroconversion of anti-Puumala hantavirus antibodies, confirming the diagnosis of acute hantavirus infection (Table 1).

**Table 1:** Overview of laboratory test results including renal function, immunology testing and hantavirus serology

	Day 1 of admission	Day 4	Day 7	12 weeks after hospital discharge
Creatinine (μmol/l)	305	204		72
eGFR (ml/min/1,73 m2)	23	37		> 90
ANCA	Negative	–		–
Anti-MPO (kIU/l)	< 3.5	–		–
Anti-Pr3 (kIU/l)	< 2.0	–		–
Anti-GBM (kIU/l)	9.7	16.0		< 0.8
Anti-Puumala IgG			Positive	Positive
Anti-Puumala IgM			Positive	Negative

*eGFR* estimated glomerular filtration rate, *ANCA* antineutrophil cytoplasmic antibody, *Anti-MPO* anti-myeloperoxidase, *anti-Pr3* Anti-Proteinase 3, *anti-GBM* Anti-glomerular basement membrane

## Discussion

This case portrays a diagnostic dilemma, with drastic therapeutic consequences of possibly starting plasmapheresis and immunosuppressants. The measurement of anti-GBM antibodies, antibodies against the α3 chain of collagen IV in the glomerular basement membrane, is considered to be highly specific and a crucial step in the work-up of patients with acute renal failure.

We used fluoroenzymeimmunoassay (FEIA) to measure anti-GBM IgG antibodies, using human recombinant α3 chain of collagen IV, with reference values, negative: < 7 U/ml; equivocal: 7–10 U/ml; positive: > 10 U/ml [4]. The specificity for detection of anti-GBM glomerulonephritis is shown to be 100%, with a sensitivity of 94.7% [5].

However, De Joode et al. described two patients with microscopic poly-angiitis (MPA), in which FEIA was also positive for both anti-GBM antibodies and anti-myeloperoxidase (MPO) antibodies [6]. These cases did not develop anti-GBM glomerulonephritis and the anti-GBM findings were considered false positives in these anti-MPO positive patients. The authors do not differentiate between wrongfully measured, or wrongfully produced antibodies and in these two cases there could be an innocent bystander effect. Considering the damage that MPA causes to the glomerular basement membrane and the similarities between the MPA and anti-GBM glomerulonephritis it is a reasonable possibility that in their case the anti-GBM antibodies were a legitimate antibody production.

In our case the increase in anti-GBM antibodies coincided with the acute phase of the hantavirus infection and it declined when renal function was restored. Combined with the specificity of the test this may represent a genuine anti-GBM antibody production.

Specific antibodies against the NC1 domain of the α3 chain of type IV collagen have not been described before in patients with hantavirus infection. However, Billheden et al. documented two cases of positive IgM antibodies against crude human GBM (*non-Goodpasture glomerular basement membrane)* in patients with hantavirus infection [7].

The same author presented a series of 47 patients with hantavirus infection of whom 77% were positive for antibodies against *non-Goodpasture glomerular basement membrane* [8]. In this series none of the patients were positive for IgM antibodies against the NC1 domain of the α3 chain of type IV collagen.

We propose three theories that could be part of the pathophysiology of anti-GBM antibody production in hantavirus infection.

First, basement membrane injury could possibly lead to production of anti-GBM antibodies.

Unfortunately, no renal biopsy was done in our case to show signs of glomerular basement membrane injury or signs of anti-GBM glomerulonephritis. It has been shown that initiating events like infection, ischemia, or toxins, can initiate an autoimmune process by damaging the glomerular basement membrane resulting in anti-GBM glomerulonephritis [9, 10].

Renal biopsies have pointed to multiple causes of renal damage in hantavirus infection; There is no evidence of direct damage to the endothelial cells [11], suggesting that the severe proteinuria caused by damage to the barrier is due to cytokine release and not by direct cytotoxicity. Cell-to-cell contact of epithelial and endothelial cells is shown to be disturbed, probably by direct infection of the epithelial and endothelial cells and podocytes [12]. Acute tubulointerstitial nephritis, with interstitial oedema and inflammatory cell infiltration has commonly been found, leading to mild to moderate interstitial, glomerular and tubular injury and extensive interstitial haemorrhage in the outer [13, 14].

Electron microscopy has shown that there is possible splitting of the tubular basement membrane [15]. The α3 chain of type IV collagen is besides in glomerular basement membrane, also found in segments of the distal tubules of the kidney [16]. Therefore damage of the distal tubules could possibly expose the NC1 domain of the α3 chain of type IV collagen and thereby causing an autoimmune reaction to the basement membrane collagen. Also, podocyte effacement is seen in hanta virus infection [17].

As described before, De Joode et al. [6] documented two patients with anti-GBM antibodies in microscopic polyangiitis. Even though they considered it false positives, the damage that MPA does to the basement membranes in the kidney could be a trigger to produce anti-GBM antibodies.

Second, Marina Garcia et al. demonstrated that hantavirus infection can cause an immense plasmablast response [18]. Immunoreactivity analysis showed that some of the plasmablasts are virus-specific, but a significant increase of reactivity was seen against virus-unrelated antigens. This might be a possible bystander effect of polyclonal B-cell activation and may be another explanation for the elevated anti-GBM. The disappearance of the antibody with the concomitant recovery of the patient fits this hypothesis.

Finally, some viruses carry epitopes that show homology to autoantigens in autoimmune diseases, called molecular mimicry. It is suggested that in some cases viral infection may lead to autoimmunity [19]. Possibly, Puumala virus carries an epitope similar to the α3 chain of collagen IV which could possibly explain the positive laboratory test. However, mimicry between GBM collagen and hantavirus is not established and a recent study looking for recognition of microbial antigens in sera positive for anti-GBM revealed only bacterial candidates [20].

## Conclusion

In summary, this is the first report of production of antibodies against the NC1 domain of the α3 chain of type IV collagen in hantavirus infection, without development of anti-GBM glomerulonephritis.

During the initial evaluation of our patient with acute renal failure, erythrocyturia and massive proteinuria, this lead to a diagnostic dilemma in which important therapeutic consequences had to be considered*.*

The anti-GBM antibodies production seems to be genuine, considering the specificity of the test and the course of disease, combined with hantavirus antibody titers.

Considering the gravity of this dilemma, awareness of the possibility of anti-GBM antibodies production in hantavirus infection is of crucial importance.

## Abbreviations

AAV: Antineutrophil cytoplasmic antibody associated vasculitis
ALT: Alanine Aminotransferase
ANCA: antineutrophil cytoplasmic antibody
anti-GBM: anti glomerular basement membrane
anti-MPO: anti myeloperoxidase
AST: Aspartate Aminotransferase
FEIA: fluoroenzymeimmunoassay
GGT: Gamma-glutamyltransferase
HFRS: Haemorrhagic fever with renal syndrome
MPA: Microscopic polyangiitis
NC1: Noncollagenous-1
NE: Nephropathia epidemica
